# The Impact of the Overall Radiotherapy Time on Clinical Outcome of Patients with Nasopharyngeal Carcinoma; A Retrospective Study

**DOI:** 10.1371/journal.pone.0151899

**Published:** 2016-03-31

**Authors:** S. D. Stoker, R. Fles, C. Herdini, F. J. F. Rijntjes, M. Tjokronagoro, S. R. Dwidanarti, K. Sikorska, C. R. Leemans, M. K. Schmidt, A. Al-Mamgani, M. A. Wildeman, S. M. Haryana, S. R. Indrasari, I. B. Tan

**Affiliations:** 1 Department of Head and Neck Surgery and Oncology, The Netherlands Cancer Institute, Amsterdam, The Netherlands; 2 Department of Otolaryngology—Head and Neck Surgery, VU University Medical Centre, Amsterdam, The Netherlands; 3 Department of Otolaryngology—Head and Neck Surgery, Gadjah Mada University, Dr. Sardjito hospital, Yogyakarta, Indonesia; 4 Department of Radiotherapy, Gadjah Mada University, Dr. Sardjito hospital, Yogyakarta, Indonesia; 5 Department of Bioinformatics, The Netherlands Cancer Institute, Amsterdam, The Netherlands; 6 Department of Psychosocial Research and Epidemiology, The Netherlands Cancer Institute, Amsterdam, the Netherlands; 7 Department of Molecular Pathology, The Netherlands Cancer Institute, Amsterdam, the Netherlands; 8 Department of Radiotherapy, The Netherlands Cancer Institute, Amsterdam, The Netherlands; 9 Department of Otorhinolaryngology, Academic Medical Centre, Amsterdam, The Netherlands; 10 Department of Histology, Cell and Tumour Biology, Faculty of Medicine, Gadjah Mada University, Yogyakarta, Indonesia; 11 Department of Oral and Maxillofacial Surgery, Academic medical Centre, Amsterdam, The Netherlands; Taipei Medical University, TAIWAN

## Abstract

**Purpose:**

In Yogyakarta, nasopharyngeal carcinoma (NPC) shows a poor response to radiotherapy treatment. Previous study showed a prolonged overall treatment time (OTT), due to interruptions during treatment. This study explores the association between clinical outcome and OTT. Secondary, the relation between clinical outcome and disease stage, waiting time to radiation (WT) and chemotherapy schedule was explored.

**Methods:**

In this retrospective cohort, 142 patients who started curative intent radiotherapy for NPC between March 2009 and May 2014, with or without chemotherapy, were included. The median follow up time was 1.9 years. Data was collected on WT, OTT, disease stage, and chemotherapy schedule. Time factors were log-transformed. Clinical outcome was defined as therapy response, loco-regional control (LRC), disease free survival (DFS) and overall survival (OS).

**Results:**

The median WT was 117 days (range 12–581) and OTT was 58 days (43–142). OTT and disease stage were not associated to any of the clinical outcome parameters. The log- WT was associated to poor therapy outcome (HR 1.68; 95% ci: 1.09–2.61), LRC (HR 1.66; 95% ci: 1.15–2.39), and DFS (HR 1.4; 95% ci: 1.09–1.81). In the multivariable analysis, significant hazard risk for poor therapy response, LRC, DFS and OS were seen for patients who didn’t received concurrent chemotherapy.

**Conclusion:**

Not receiving concurrent chemotherapy showed the strongest risk for poor outcome. Since the choice of chemotherapy is related to a variety of factors, like the WT and patient’s physical condition when radiation can start, careful interpretation is needed. Reason for not finding a relation between OTT and clinical outcome might be the low number of patients who finished radiotherapy within 7 weeks, or by a stronger detrimental effect of other factors.

## Introduction

Nasopharyngeal cancer (NPC) is the most common malignancy in the head and neck region in Indonesia [1]. The incidence is estimated at 6:100 000, which is probably an underestimation since many patients living in the rural areas might stay undiagnosed [2]. In literature, 3- year overall survival for NPC are reported as 70 to 80%, or even higher. Studies in Indonesia showed a 3-year overall survival of only 30% [3, 4]. Most of the studies with the high survival rates are derived from clinics with advanced and readily available treatment facilities. More than 85% of the NPC patients are diagnosed in low- and middle-income countries, with less advanced equipment and limited capacity [2, 5]. Therefore, actual NPC survival will be much lower than reported in literature and more likely to be in the range of Yogyakarta. This study aims to identify factors associated with poor clinical outcome.

The best treatment for advanced NPC is radiotherapy in combination with chemotherapy, 60–70 Gray (Gy) to the nasopharynx and neck metastases and an elective dose of 40–50 Gy to the uninvolved parts of the neck [3, 6]. For stage I (AJCC 6th edition), radiotherapy alone gives satisfactory results [7–9]. General consensus for radiotherapy is that treatment should be given without interruptions. When treatment is interrupted repopulation of tumour cells can occur, which is believed to be a significant risk for treatment failure [10–13]. This phenomenon has been proven in both xenograft animal models and clinical studies with cervical cancer, bladder cancer and head and neck cancer [13–19]. One of the largest studies in head and neck cancer concerning the effect of overall radiotherapy treatment time (OTT) on clinical outcome is from the Danish group [20]. They proved the benefit of 6 fractions a week above 5 fractions per week. Unfortunately, NPC was excluded.

It is assumable that treatment interruption during radiotherapy in NPC will also negatively affect clinical outcome, but multiple, large studies confirming this, are lacking. Besides, controversial results are found [17, 21–27]. One of the most recently published studies is from Li et al. [27]. They included 321 patients treated with radiotherapy for NPC, but they couldn’t proof the negative effect of a long OTT on local-regional control and distant metastases free survival (Li 2015). Another way to shorten OTT is by using accelerated radiation schemes. However, also in these studies conflicting results are shown with regard to the impact on outcome [24, 25–26]. Pan et al. found improved loco-regional control (LRC) and overall survival (OS) [25], but Lee et al. and Theo et al. only showed more side effects without improved clinical outcome [24, 26].

Previous study in Yogyakarta showed a mean number of missed days of 10 per patient. Meaning that to complete the full course of treatment, the OTT was prolonged by two weeks, since radiotherapy is only given on weekdays [28]. In the discussion was hypothesized that the interruptions during radiotherapy, and thus the length of the OTT, could have influenced clinical outcome to a great extent. Therefore, the aim of the current study is to explore the association between OTT and clinical outcome in NPC. If these results show a significant disadvantage in clinical outcome when the OTT is longer, a first step is made in finding solutions for the problem of the poor prognosis of patients with NPC. Since, the OTT can be shortened if effort is made [28]. It is likely that clinical outcome is also related to disease stage, waiting time to start radiotherapy (WT), and the chemotherapeutic schedule (neo-adjuvant and/or concurrent), therefore these factors were also taken in analysis.

The preferred treatment of choice for NPC in this institute is concurrent chemo radiotherapy. For many patients it was not exceptional that the waiting time for radiotherapy exceeded 3 months. To prevent disease progression in this period, neo-adjuvant chemotherapy was given. In some patients the physical condition was deteriorated to such extend that another course of chemotherapy was not an option anymore, so they received radiation without chemotherapy.

## Method

### Study design and eligibility

This retrospective study was conducted in Dr. Sardjito hospital, Gadjah Mada University, in Yogyakarta, Indonesia. Ethical approval was obtained from this institution’s review board. Data was obtained from the hospital’s medical records and the online data-management system introduced in 2008 [29]. Patient information was anonymized and de-identified before analysis. Patients were deemed eligible when they started curative intent radiotherapy, with or without chemotherapy, for histologically confirmed NPC (World Health Organization type 1, 2 or 3). There is an overlap of patients with the studies published in 2013 and 2014 [4, 28]. There were no medical intervention in these or the current study, therefore no separate analysis was performed.

Between March 2009 and May 2014, 193 patients started radiotherapy for NPC. Fifty-one patients (26%) were excluded. Reasons for exclusion were; drop out during radiotherapy (due to side effects (n = 12), death (n = 5), financial problems (n = 4) or unknown reason (n = 8)), suspect distant metastasis (n = 9), unreliable staging (n = 7), second primary (n = 1), or a short (<3 months) post-treatment follow up period (n = 5). Subsequently, 142 patients were included for study outcome analysis. Staging at diagnosis was performed by computed tomography (CT) scan of the head and neck, ultrasound of the abdomen, X-ray of the thorax and a bone survey, and scored according to the 6th edition of the American Joint Committee on Cancer (UICC/AJCC).

### Treatment

All patients were treated with external beam radiotherapy, 2 dimensional or 3 dimensional, in fractions of 2 Gy per day, 5 times a week (not in the weekends). The protocol for NPC was 60 Gy for T1 disease and 66–70 Gy for T2-4 to the nasopharynx; and 40–46 Gy for N0-1 and 50 Gy for N2-3 to the whole neck (including the supraclavicular fossa). Persistent nodes were radiated up to 66–70 Gy. The overall treatment time (OTT), starting from the first day of radiotherapy until the last day of radiotherapy, could be finished within 42–47 days. The waiting time to radiotherapy (WT) is calculated from the day of diagnosis until start radiotherapy.

Different schedules for chemotherapy were used. The schedule depended on the patient’s physical status, type of insurance, availability of the drugs and waiting time for radiotherapy at that time. When the waiting time to radiotherapy was long (in particular in case of poor patients), neo-adjuvant chemotherapy was given to prevent progression. Concurrent chemotherapy was the preferred choice, but physical deterioration made this schedule not possible for all patients. Neo-adjuvant chemotherapy consisted of; cisplatin, carboplatin, fluorouracil, capecitabine, docetaxel, or paclitaxel. Concurrent chemotherapy regimens consisted of weekly cisplatin or carboplatin. Patients were categorized into 4 groups; concurrent, concurrent and neo-adjuvant, neo-adjuvant or no neo-adjuvant or concurrent chemotherapy. In five cases, adjuvant chemotherapy was given. Adjuvant chemotherapy consisted of carboplatin, paclitaxel and/or capecitabine. Adjuvant chemotherapy was ignored for group categorization.

### Clinical outcome

Clinical outcome was measured by 4 parameters, i.e.; therapy response, local-regional control (LRC), disease free survival (DFS) and overall survival (OS).

#### Therapy response

Post-treatment protocol for treatment response was endoscopy, biopsy of the primary local tumour site, CT-scan of the head and neck area, ultra-sound of the abdomen, x-ray of the thorax and a bone survey. Unfortunately, not all patients completed this protocol. Therapy response was set as complete response or incomplete response. In case of uncertainty about therapy response, patients were excluded for therapy response analysis. Complete response was defined as; 1) all imaging studies, including biopsy showed no disease, and there was no recurrent disease within 6 months or, 2) in case of clinical examination only (including endoscopy), and there was no suspicion for disease, and no suspicion of recurrent disease in the following 2 years. Incomplete response was defined as; 1) local or regional disease confirmed by biopsy, or cytology within 6 months post-treatment or, 2) a suspect CT scan for loco-regional residual disease and died within 1 year or, 3) distant metastases within 6 months after radiotherapy, confirmed by progression during follow up. Exclusion criteria for therapy response analysis were 1) no clinical follow up in the first 6 months or, 2) suspected incomplete response (never confirmed by biopsy or progression during follow-up), but alive without complaints for at least 2 years.

#### Local-regional control

LRC was defined as the time between the last day of radiotherapy till the date of local and/or regional disease. In case of persistent disease, the date of event was set at 3 days post-treatment. Patients were censored if they were alive without local-regional disease. In case of uncertainty about the local-regional status they were excluded for this analysis.

#### Disease free survival

DFS was defined as the time between the last day of radiation till the date of recurrent disease (local, regional or distant) or the date of death. In case of persistent disease, the date of event was set at 3 days post-treatment. Patients who had no evidence of disease at last follow-up were censored at the date of last contact.

#### Overall survival

OS was defined as the time between start date of radiotherapy till the date of death. A different starting point was chosen, since the length of OTT time differed considerably, which would affect OS. Patients who were alive at last follow up were censored. Patients who died due to a second primary tumour were excluded from this analysis.

### Statistical analysis

The main interest was to relate OTT and WT to therapy response, LRC, DFS and OS. These time-related predictors (OTT and waiting time) were skewed to the right, and therefore transformed into (normal) log scale. Log-transformed OTT and WT were analyzed in univariable logistic regression and Cox models. Shapes of the relationships for the time to event outcomes were evaluated using martingale residuals plots. In the multivariable models we included WT, OTT as well as chemotherapy treatment schedule and tumor stage as possible confounders. For the time to event outcomes an independence test based on log rank statistics was used to find a cut-off, which separates best patients with good and poor prognosis. The analyses were performed using SPSS software (version 22) and R software (version 3.1.0). P-values < 0.05 were considered statistically significant.

## Results

### Patients & treatment description

From the 142 patients, 97 (68%) were male. The histological type of NPC was WHO 3 in 94% of the cases. At diagnosis, 84% had late stage disease. Chemotherapy was administered in 90% of the patients (Table 1). For 6 (4%) patients there was ambiguity about the actual given chemotherapy. The median WT was 117 days (range 12–581). The median dosage was 66 Gy (range 60–76). The median OTT was 58 days (range 43–142). The median follow up time was 708 days (1.9 years) (calculated from the start of radiotherapy).

**Table 1:** Tumour stage at diagnosis & treatment.

Tumour stage	Patients	Chemotherapy schedule							
		Group 1		Group 2	Group 3		Group 4		
AJCC	Total (%)	Conc	Conc & adj	Conc & neo	Neo	Neo & adj	Adj	No chemo	Missing
I	3 (2.1)	0	0	0	0	0	0	1	2
IIa	1 (0.7)	0	0	0	1	0	0	0	0
IIb	16 (13)	6	0	2	5	1	0	4	1
III	50 (35)	19	0	6	23	0	0	1	1
IVa	20 (14)	6	0	0	11	0	2	0	1
IVb	49 (35)	9	1	6	29	0	1	2	1
Total (%)	142 (100)	40 (28)	1 (0.7)	14 (10)	69 (49)	1(0.7)	3 (2.1)	8 (5.6)	6 (4)

AJCC = American joint Committee for Cancer 6^th^ edition; conc = concurrent chemotherapy; neo = neo-adjuvant chemotherapy; adj = adjuvant chemotherapy

### Association between OTT and outcome

#### Therapy response

**Fig 1:**
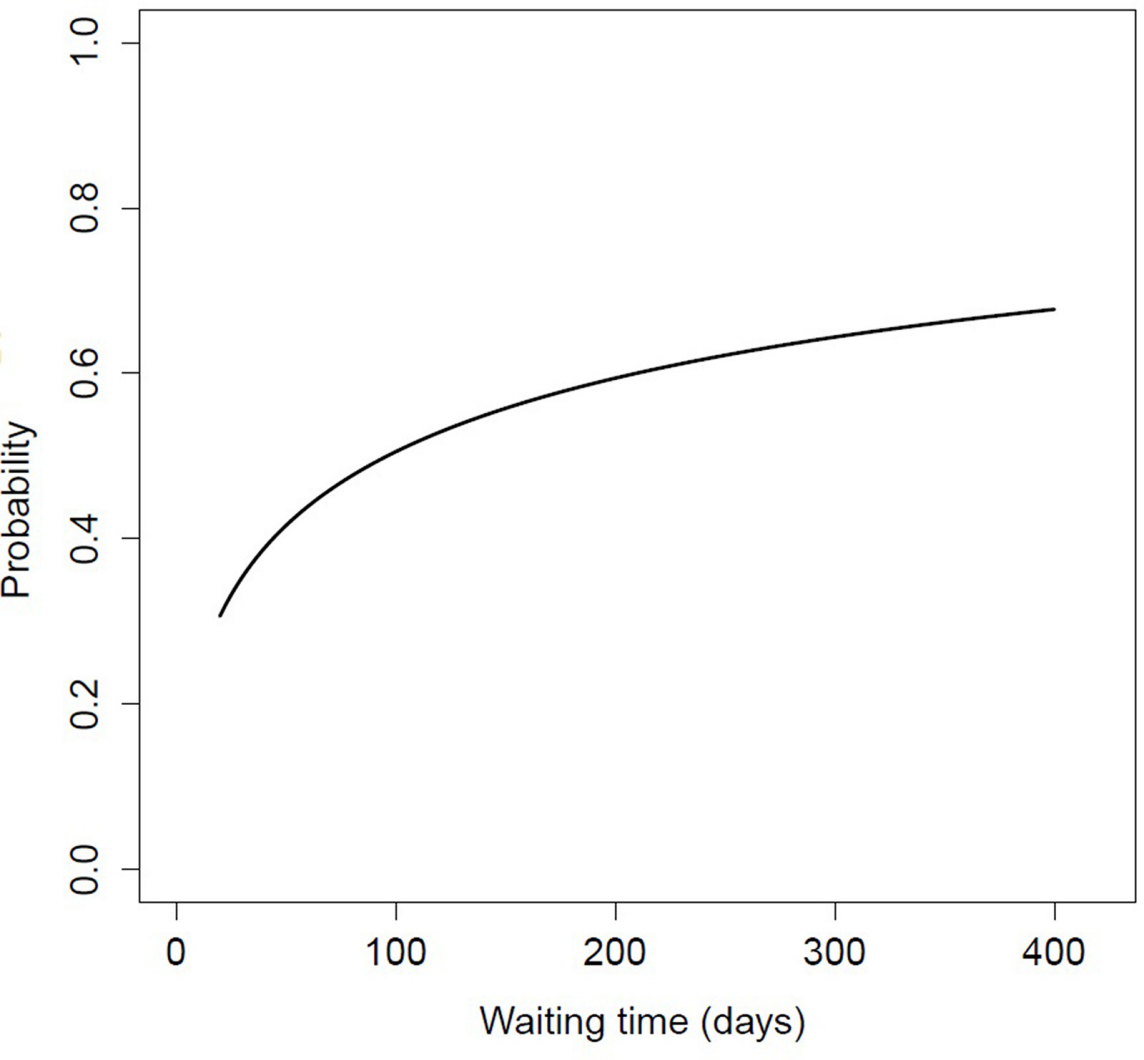
Probability of incomplete response as function of the waiting time (WT). Probability of 0 is a complete response and 1.0 an incomplete response. The WT is shown as transformed back into a continuous scale. In the first 3 months, the increase of probability of incomplete response is steeper than when the length of WT is longer. The probability on incomplete response seems to reach a plateau level.

Therapy response was available for 120 patients, 60 patients had a complete response. Disease stage was not associated to response. Patients with a complete response had an equal median OTT as patients with an incomplete response, 58 days (Table 2). The median WT was 101 versus 140 days for patients with a complete or incomplete response, respectively (Kruskal-Wallis p<0.01) (Table 2). Also in the univariable analyses, wherein time parameters were transformed into a (normal) log scale, significant odds ratios for poor therapy outcome were found for log WT, and not for log OTT (Table 3). A plot summary was made to visualize the clinical interpretation of the increased risk when the WT is increased (Fig 1). In the multivariable analysis for therapy response, only chemotherapy schedule remained significant (Table 4).

**Table 2:** OTT & WT and complete or incomplete therapy response.

	Total	Complete response	Incomplete response	
	Days (range)	Days	Days	Mann Whitney U
**Median OTT**	58 (43–142)	58	58	p = 0.26
**Median WT**	117 (12–581)	101	140	p = 0.01*

OTT = overall radiation treatment time; WT = waiting time to radiotherapy

*significant outcome

**Table 3:** Univariable logistic regression (therapy response), and Cox model (LRC, DFS and OS).

	Therapy response		LRC		DFS		OS	
	OR	P-value	HR	P-value	HR	P-value	HR	P-value
**Log OTT**	2.3	0.37	1.4^1^	0.49	1.2	0.73	2.0	0.21
**Log WT**	1.7	0.02*	1.7	0.01*	1.4	0.01*	1.3	0.09

OR = odds ratio; HR = hazard ratio; LRC = loco-regional control; DFS = disease free survival; OS = overall survival; OTT = overall radiation treatment time; WT = waiting time to radiotherapy

1 = based on a cut-off point of 72 days

*significant outcome

**Table 4:** Multivariable analysis.

	Therapy response		LRC		DFS		OS	
	OR	P-value	HR	P-value	HR	P-value	HR	P-value
**Log OTT**	3.6	0.22	1.5^1^	0.39	1.4	0.52	2.0	0.27
**Log WT**	1.0	0.98	1.1	0.87	1.0	0.92	0.9	0.67
**Chemotherapy**								
Concurrent	1.0		1.0		1.0		1.0	
Conc & neo-adj	0.9	0.90	1.0	0.98	1.0	0.991	0.4	0.29
Neo-adjuvant	6.4	0.01*	4.4	0.03*	3.0	<0.01*	3.1	0.02*
None	6.1	0.03*	3.8	0.04*	2.5	0.07	2.4	0.19
**Tumor stage**								
IVb	1.0		1.0		1.0		1.0	
IVa	0.9	0.90	1.0	0.99	1.0	0.99	0.7	0.34
III	1.0	0.98	1.0	0.90	0.9	0.68	0.5	0.02*
IIb	1.8	0.39	1.3	0.64	1.0	0.89	0.6	0.23
IIa	0.6	0.72	0.5	0.59	0.4	0.36	0.3	0.31

OR = odds ratio; HR = hazard ratio; LRC = loco-regional control; DFS = disease free survival; OS = overall survival; OTT = overall radiation treatment time; WT = waiting time to radiotherapy

*significant outcome

1 = based on a cut-off point of 72 days

#### Local regional control

**Fig 2:**
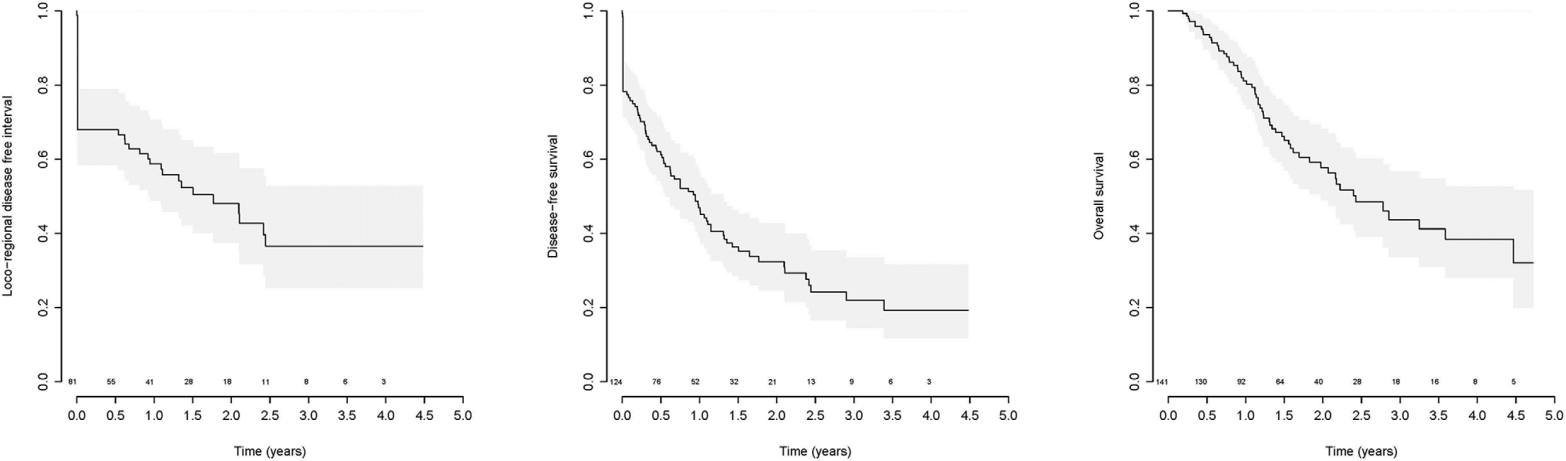
Survival plots. (a) local regional control, (b) disease free survival and (c) overall survival. Above the x-axis is the number of patients at risk.

**Fig 3:**
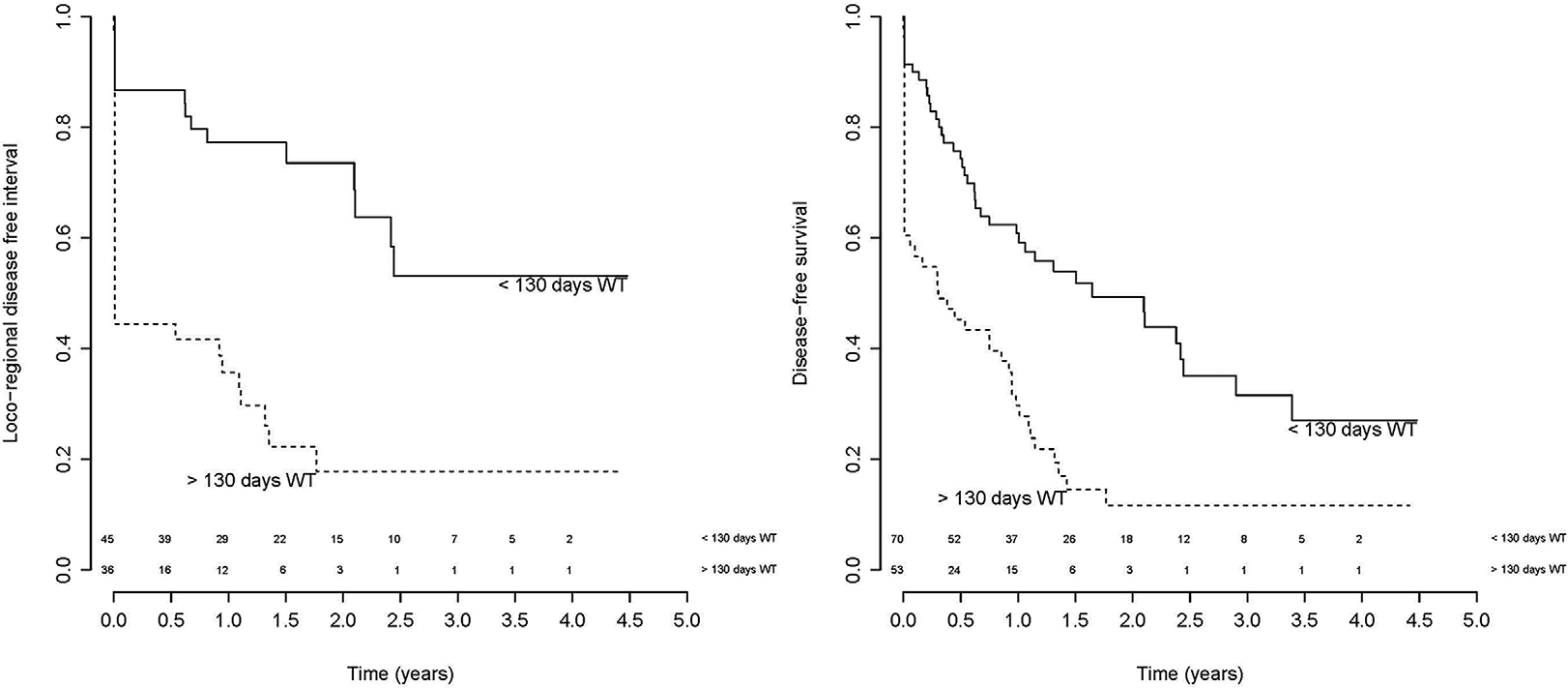
Survival plots, stratified for waiting time. (a) local-regional control and (b) disease free survival stratified for a waiting time longer or shorter than 130 days (log rank for both plots <0.001). Above the x-axis is the number of patients at risk.

**Fig 4:**
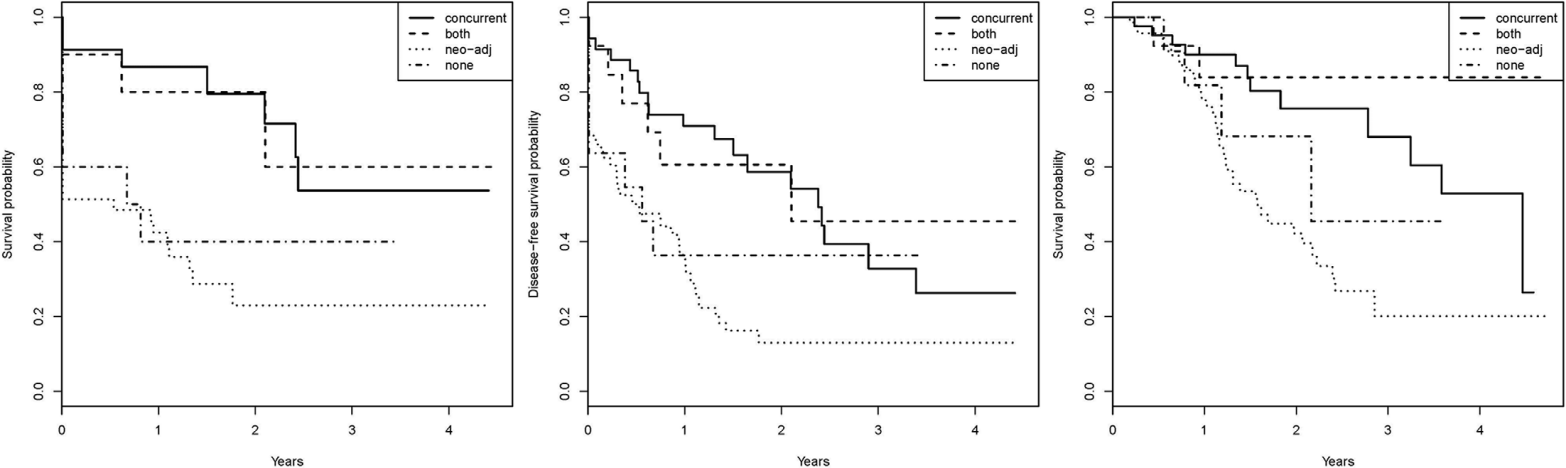
Survival plots, categorized per chemotherapy schedule. (a) local regional control, (b) disease free survival and (c) overall survival.

For LRC analysis, 81 patients were available, 43 events occurred. The 2-year LRC was 48% (median 1.77 years) (Fig 2A). Univariable Cox model showed a significant HR for poor LRC when the log WT was increased. Based on the exploratory analysis the association of log OTT and LRC was not linear. The optimal cut-off point was selected for 72 days, however this was statistically not significant (log rank p-value 0.49) (Table 3). A significant optimal cut-off point for the WT, which separates good and poor LRC, was found at 130 days (Fig 3A, log rank p<0.001). In the multivariable Cox-model only chemotherapy remained significant (Table 4, Fig 4A).

#### Disease free survival

For DFS analysis, 124 patients were available, 86 events occurred. The 2-year DFS was 32% (median 0.94 years) (Fig 2B). Univariable Cox model showed a significant HR for poor DFS when the log WT was increased. Log OTT was not significantly associated to DFS (Table 3). A significant optimal cut-off point for the WT, which separates good and poor DFS, was found at 130 days (Fig 3B, log rank p<0.001). In the multivariable Cox-model only chemotherapy schedule remained significant; patients who only received neo-adjuvant chemotherapy had the poorest DFS (Table 4, Fig 4B).

#### Overall survival

For OS analysis 141 patients were available, 59 events occurred. The 2-year OS was 58% (median 2.4 years) (Fig 2C). Univariable Cox model didn't show association between the probabilities of poor survival and log OTT or log WT (Table 3). In the multivariable Cox-model chemotherapy schedule and disease stage remained significant; patients who only received neo-adjuvant chemotherapy or who had stage III disease had the poorest OS (Table 4, Fig 4C).

## Discussion

The current study showed no correlation between overall treatment time and clinical outcome in patients with NPC. It was expected that a prolonged OTT was related to unfavourable clinical outcome, based on several studies that confirmed the benefit on tumour control and survival when radiotherapy for head and neck cancer was given without interruptions [13, 16–20]. Nevertheless, studies confirming this effects for NPC are limited [17, 21–23, 27]. Some studies focussed on the benefit of accelerated schedules of NPC, but also these results are conflicting [24, 25]. The current study analysed 142 patients with NPC only, all treated with curative intent for primary NPC. Therefore it was expected to be of great value for estimating the impact of an interrupted radiotherapy treatment in NPC. Besides, it could proof the focus for improving the clinical outcome of NPC patients.

Clinical outcome was defined in 4 parameters i.e.; treatment response, LRC, DFS and OS. This was based on experience from previous studies where we found difficulties completing follow up data [4, 30]. For every analysis, some patients had to be excluded due to uncertainty. Not only because of deviation in the follow up protocol, but also due to difficulties in estimating disease status. For therapy response, the combination of imaging studies and clinical follow up was used. LRC was the preferable outcome parameter, because the given treatment was primarily a loco-regional treatment. Unfortunately, only 81 (57%) patients could be analysed for LRC, due to the lack of local-regional status data. DFS also includes patients who died without information about their disease stage. It is assumable that these patients died because of recurrent disease, therefore DFS was also a valuable outcome parameter. OS gave a good impression about the general prognosis for these patients. Except for one, all patients could be analysed for OS.

Unexpectedly, our results did not show an increased risk for poor clinical outcome when OTT was prolonged. Although all odds and hazard ratios were >1, no significance was reached. Based on these results it would be short-sighted to conclude that OTT does not affect therapy outcome for NPC. Studies that showed the increased risk on local failure in head and neck cancer when OTT was prolonged used a cut-off point between 7–8 weeks [21]. Also these cut-off points (49 days or 56 days) did not show significant increased risk (data not shown). This might be explained by the low number of patients who finished treatment within the recommended 7 weeks (n = 16). An explanation for not finding increased risk ratios in the continuous scale analysis might be that the effect of missing 2–4 weeks is not worse than missing 1–2 weeks. Another explanation could be that the OTT was overruled by other factors, and that the sample size was too small. As also concluded by others, larger, multi-institutional studies are needed to estimate the safe range OTT for NPC [27].

Another remarkable finding was that disease stage at diagnosis was not related to clinical outcome. The long waiting time can be an explanation. The negative effect of long WT is frequently described [31]. In the current study the median delay to initiation of radiation treatment was 4 months. The probability of an incomplete response, depending on waiting time, had the shape of a log-function (Fig 1). This means, in the first months it is of great importance to prevent delay, but at a certain time, the probability of poor outcome reaches a maximum despite a longer WT. Many patients received neo-adjuvant chemotherapy to overcome the waiting time. Nevertheless, disease progression and deterioration of the physical condition in this period is inevitable. The disease stage at diagnosis might not be representative for the actual stage that is treated. Some patients might have developed distant metastasis, which affects prognosis tremendously. In a currently on-going study we are evaluating the disease progression during the WT. Patients with a WT exceeding 3 months will be re-staged. Besides better insight in the actual disease stage, patients who develop stage IVc can be prevented from treatment that is deemed unsuccessful and has a high morbidity. Unnecessary use of the treatment units can be prevented, which will decrease WT for other patients. In that on-going study, also the physical deterioration during the WT will be analysed. A poor physical condition is frequently related to poor treatment outcome [18, 19].

Another shortcoming in disease stage are the methods used for confirming disseminated disease. Ultra sound, x-ray of the thorax and bone survey (x-ray of the bones) have low sensitivity for bone and lung metastasis. PET-scan was not available and bone scintygraphy is only used (and covered by insurance) in case of suspected bone lesions on the bone survey. Therefore, the presence of distant metastases might have been underestimated.

The group of patients that didn’t receive concurrent chemotherapy had significant worse clinical outcome. This factor remained significant for all outcome parameters in multivariable analysis. Also here careful interpretation is needed. Several other studies found the beneficial effect of concurrent chemo-radiotherapy above neo-adjuvant chemotherapy [32, 33]. Nevertheless, our results are probably biased by the choice of treatment. In general, patients who received concurrent chemotherapy are either ‘poor’ patients in a good condition after neo-adjuvant chemotherapy to overcome the waiting time, or ‘rich’ patients with a short waiting time who can start concurrent treatment immediately. The deterioration of the physical condition by neo-adjuvant treatment made concurrent chemotherapy too toxic for many patients.

While waiting for expanding the radiation facilities, other treatment modalities are needed to overcome the waiting time. These modalities should prevent progression, without deterioration of the patient’s condition and remain the possibility of concurrent chemo-radiotherapy, since concurrent chemo-radiotherapy treatment modality has been shown repeatedly as the most effective for advanced NPC [32].

The 2-year overall survival was 58%. When interpreting these statistics please note that patients who did not finish radiotherapy treatment were excluded from analysis, and therefore better than the rate reported in the introduction [4]. During the waiting time and during treatment patients died or dropped out, which would have had affected the overall survival negatively. The reason for excluding these patients in the current study was the different study aim.

In conclusion, although OTT and the WT are probably of influence on clinical outcome, this study showed the strongest effect for chemotherapy schedule. Every patient with advanced stage disease should get concurrent chemo-radiotherapy [33]. Only by reducing the waiting time, this can be accomplished. Therefore, radiation facilities should be expanded. However this is a time consuming process, and will not help to improve the life of cancer patients at short notice. While waiting for more units, more effectively use of the already available machines can be a step forward, like longer operational hours and better maintenance to prevent technical problems. Also, alternative (new) treatment modalities to overcome the waiting time, which prevent progression and avoid physical deterioration, might be helpful to remain the possibility to give concurrent chemotherapy.

Recently, problems have only increased. In the study period, the median waiting time for radiotherapy was 4 months. Due a change in the insurance system since January 2014, aiming to provide national health care insurance for all inhabitants of Indonesia, nowadays the waiting time for radiotherapy exceeds 1,5 year. Eighty-five per cent of the NPC patients are diagnosed in low-income countries, therefore Indonesia is not the only country who is struggling [1,5]. This is confirmed in a recently published article that showed the shortfall of radiotherapy capacity by country [34].
